# Extracting Superficial Scattering by Q‐Sensing Technique

**DOI:** 10.1002/jbio.202400262

**Published:** 2024-09-06

**Authors:** Alon Tzroya, Hamootal Duadi, Dror Fixler

**Affiliations:** ^1^ The Faculty of Engineering and the Institute of Nanotechnology and Advanced Materials Bar Ilan University Ramat Gan Israel

**Keywords:** Monte Carlo simulations, polydisperse, polarization, Q‐sensing, scattering, Valery V. Tuchin

## Abstract

Optical properties determine how light interacts with biological tissues. The current methods for measuring these optical properties are influenced by both deep and superficial skin layers. Polarization‐based methods have been proposed in order to determine the influence of deep layer scattering. Polarized light allows for the separation of ballistic photons from diffuse ones, enhancing image contrast and resolution while providing additional tissue information. The Q‐sensing technique captures co‐polarized $\left(I_{∥}\right)$ and cross‐polarized $\left(I_{⊥}\right)$ signals, making it possible to isolate the superficial scattering. However, the random structure of tissues leads to rapid depolarization of the polarized light. Detecting where the light becomes depolarized aids in sensing abnormalities within the tissues. Hence, this research focuses on identifying where depolarization occurs within the tissue. Tissue‐mimicking phantoms, simulating the optical properties of biological tissues, are created to measure depolarization at various thicknesses. Experimental findings are validated with a Monte Carlo simulation, modeling polarized light behavior through the polydisperse tissue (as the tissue scatterers are heterogeneous in size). Additionally, the research demonstrates how polarized light can extract the optical properties of the medium.

## Graphical abstract

Exploring the use of polarized light by Q‐sensing technique to enhance sensing of abnormal situations in tissue. By isolating superficial scattering from deeper layers, identifying areas of depolarization is possible, allowing the detection of tissue abnormalities and extraction of superficial scattering. The experimental results are supported by Monte Carlo simulations demonstrate the potential of polarized light sensing.

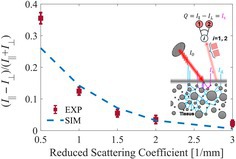

## 1 Introduction

Polarized light has proven to be highly sensitive to medium structures and thus been widely adopted in optical imaging to probe microstructural features inside biological tissues [1]. Moreover, for tissues, their randomness in structure causes rapid depolarization of the polarized light [2, 3]. However, in certain tissues and cell structures, the degree of polarization of transmitted or reflected light can still be measured even in thick tissues [4]. Meaning, polarized light allows one to differentiate ballistic photons from diffuse ones [5]. This property offers improved image contrast and resolution, as well as additional information about tissue structure, absorber presences, and blood supply in tissues [6]. In addition, the polarization status of the backscattered light can be measured to characterize the superficial layer of skin for cancer diagnostic purposes [7, 8]. There are also specific molecules that can be detected by utilizing polarization like glucose [9]. Furthermore, in environmental monitoring, polarized light can be used to detect pollutants such as microplastics in water by analyzing the polarization properties of scattered light [10].

Optical properties play an important role in determining how light interacts with biological tissues. These properties are essential for various applications as mentioned. Methods for extracting optical properties include techniques such as diffuse reflection, near infrared spectroscopy, optical coherence tomography and more [11, 12, 13, 14, 15]. Accurate knowledge of these properties allows for better diagnosis, targeted therapy, effective pollutant detection, and so forth. However, current methods face challenges due to complex tissue structures, multiple scattering events, penetration depth issues, and instrumental limitations [14]. Biological tissues have complex, heterogeneous structures that make it difficult to accurately measure optical properties. Hence, we proposed to utilize the polarization of light, specifically the Q‐sensing technique to extract the optical properties.

**FIGURE 1:**
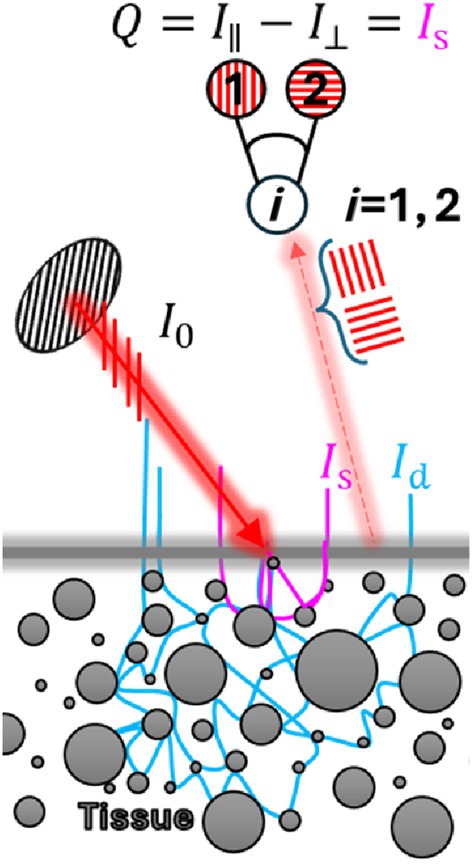
Interaction of polarized light with tissue. $I_{0}$ represents the incident polarized light, $I_{s}$ the superficial scattered light, and $I_{d}$ the diffuse light. The polarized detector distinguishes between co‐polarized and cross‐polarized light, with the Q‐sensing parameter is defined by the reduction between these elements.

In tissues, most of the backscattered light originates from the deeper layers, posing difficulties in isolating and observing the tissue's superficial layer [7]. Thus, by capturing co‐polarized $\left(I_{∥}\right)$ and cross‐polarized $\left(I_{⊥}\right)$ signals using a linear polarizer, it is possible to isolate the superficial scattering of the photons that are still polarized from the background diffuse light [16]. The co‐polarized signal consists of a superficial reflectance of polarized light $\left(I_{s}\right)$ and half of the background is diffuse light from deep layer multiply‐scattered photons $\left(I_{d}\right)$, while the cross‐polarized signal represents the other half [17, 18]. Figure 1 demonstrates how polarized light interacts with a tissue. An incident polarized light beam, $I_{0}$, enters the tissue, resulting in $I_{s}$ and deeper diffused light, $I_{d}$. The detector captures both $I_{∥}$ and $I_{⊥}$, allowing differentiation between superficial and deep scattered light. The blue lines represent the paths of diffused light within the tissue, while the red region indicates the initial light cone.

Therefore, the Q‐sensing effectively returns the normalized scattered intensity of the superficial layer as follows [17]:

(1) $$I_{∥}=I_{s}+\frac{I_{d}}{2};I_{⊥}=\frac{I_{d}}{2}$$

The total intensity (*I*) is defined by:

(2) $$I=I_{∥}+I_{⊥}=I_{s}+I_{d}$$

while the *Q* parameter is calculated as follows:

(3) $$Q=I_{∥}-I_{⊥}=I_{s}$$

In order not to be affected by variable factors such as the intensity of the light source, the position of the detector and environmental changes, we normalize the *Q* value with the total intensity:

(4) $$Q^{\prime }=\frac{Q}{I}=\frac{I_{∥}-I_{⊥}}{I_{∥}+I_{⊥}}=\frac{I_{s}}{I_{s}+I_{d}}$$

where $Q^{\prime }$ is the normalized *Q* parameter, indicating the normalized scattered intensity from the superficial layer. Both I and Q parameters associate with the Stokes vector, which is a known representation of the polarization state of light as $S={\left[I\;Q\;U\;V\right]}^{T}$, *Q* and *U* represent the intensity differences between orthogonal linear polarization and *V* represents the difference between left and right circular polarizations [6]. Our research show the depolarization of light within a tissue by measuring $I_{∥}$ and $I_{⊥}$ for varying phantoms and different layer thicknesses. We then compare the experiment to a Monte Carlo (MC) simulation and show good agreement. We present the dependence of the *Q* parameter on the layer thickness and how we can utilize it to measure the optical properties of thick scattering samples.

## 2 Materials and Methods

### 2.1 Optical Setup

The optical setup includes an *XYZ* translation stage (PT3, Thorlabs) for precise alignment of the photodiode within the illumination spot. Optimal alignment is achieved when the detector registers the maximum $I_{∥}$.This is defined in our system when the output polarization, determined by the linear polarizer (LPVISE100‐A, Thorlabs) (P2), which is coupled to the detector and is set parallel to the light source polarization, determined by the other linear polarizer (LPVISE100‐A, Thorlabs) (P1), at specific *XYZ* coordinates. The translation stage is slowly adjusted along the *X* and *Y* axes to find a maximum intensity in that respective axis. After this calibration, the stage is set to the *XY* coordinate that maximizes the intensity of both. Then adjusting the detector height is the final step of the calibration that ensures proper lens alignment within the *XY* coordinates and accurate intensity measurement.

**FIGURE 2:**
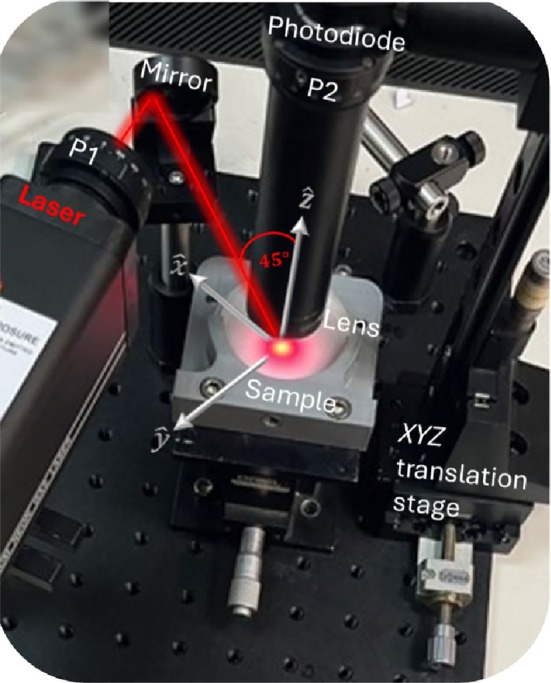
Ne–He laser illuminates the sample through the first polarizer P1 where the light is reflected from the mirror at $45^{{}^{\circ}}$ angle. The reflected light is gathered by the photodiode using a lens to enhance its sensitivity. The intensities $I_{∥}$ and $I_{⊥}$ are measured by rotating the second polarized P2 from the horizontal position $90^{{}^{\circ}}$ to the perpendicular polarization.

A continuous Ne‐He laser (HNLS008R, Thorlabs) with a wavelength of 632.8 nm and a power output of 0.8 mW serves as the light source. The laser beam is coupled with P1 polarizer, aligned at an angle that outputs maximum intensity at the preconfiguration test. The beam is then directed toward a mirror, which deflects it at a 45° angle, relative to the *Z*‐axis. Upon hitting the phantom, the light scatters, and the reflected light is collected by a photodiode (SM05PD2B, Thorlabs) with an active area of 13 [mm^2^]. This photodiode is attached to a lens to collect light from a small area, increasing the dynamic range of the measurements. After passing through the lens, the light interacts with the P2 polarizer that can be rotated to record both $I_{∥}$ and $I_{⊥}$ intensities. The photodiode is maintained at a fixed distance from the sample surface to ensure consistent measurements. Data from the photodiode are captured by an oscilloscope, transmitted to MATLAB for analysis, and processed to extract relevant information, like the $Q^{\prime }$ parameter from Equation (4). The experimental arrangement with the beam trajectory is depicted in Figure 2.

### 2.2 Sample Preparation

The samples used in this study are liquid tissue‐mimicking phantoms containing Intralipid (IL) (Intralipid 20% Emulsion, Sigma‐Aldrich, Israel) and double distilled water. The use of IL allows for varying the scattering coefficients $\left(\mu_{s}\right)$, defined as the probability of a photon to undergo scattering event [19]. The scattering of the sample is determined by the concentration of insoluble particles, their size, and the system's geometry [19, 20]. Our tissue mimicking phantoms are in different thickness ranging from 0.6 to 1.5 [mm] with jumps of 100[μm] and between 2 and 10 mm with jumps of 1 mm, making it a total of 19 different thicknesses. We could not get thinner thickness than 0.6 [mm] due to the capillary forces on the sample making it unable to spread evenly on the petri dish. The $\mu_{s}$ parameter and anisotropy $\left(g\right)$ is calculated according to an equation known in the literature [21].

The reduced scattering coefficient $\left(\mu_{s}^{\prime }\right)$ that takes into consideration the loss of momentum of light is as follows:

(5) $$\mu_{s}^{\prime }=\left(1-g\right)\cdot \mu_{s}\left[\frac{1}{\mathrm{mm}}\right]$$

Therefore, we made five phantoms where their $\mu_{s}^{\prime }$ is ranging from 0.5 to 3 [1/mm] and measured them with different slab thicknesses, forming a total of 5×19=95 samples.

### 2.3 MC Simulations

In this study, we build a MC simulation, a well‐known method that predicts the mean behavior of complex systems by numerical computations of random events [22]. We utilized the meridian plane MC simulation to investigate the propagation of polarized light through turbid media [23]. The simulation accounts for scattering events and tracks the Stokes parameters as it interacts with the medium. The process is iterated with $10^{7}$ photons for each corresponding concentration and layer thickness in order to compare the results with the experiment.

For each sample, the incident light is initialized with a uniform weight and $45^{{}^{\circ}}$ incident angle as in the experimental setup. The light source is modeled as a pencil beam, and the Stokes vector is set to horizontal polarization $\left[1\;1\;0\;0\right].$ Photons are propagating through the medium, with their position and polarization states updated at each scattering event. The status of each photon (alive or dead) is tracked. A photon is considered dead if it is absorbed, exceed the slab thickness, or experiences more than the maximum number of $10^{3}$ scattering events [24]. In our simulation, photon propagation is performed using a random sampling approach to determine step size, according to:

(6) $$s=-\frac{\ln\left(z\right)}{\mu_{a}+\mu_{s}}$$

where *z* is a random number between (0,1]. Thus, the photon is propagated in relation to the step size:

(7) $${\hat{r}}_{\mathrm{new}}={\hat{r}}_{\mathrm{old}}+s\cdot \hat{k}$$

where $\hat{r}$ is the position of the photon, and $\hat{k}$ is the propagation vector of the photon acquired by the scattering angles θandψ [23, 25]. The required scattering describing angles, θ (scattering angle) and ψ (angle of rotation into the scattering plane), are chosen using the rejection method [23]. According to Mie theory, when a photon undergoes scattering by a spherical particle, the Mueller matrix (also known as the scattering matrix) takes the a symmetric form composed of $s_{11},s_{12},s_{33},s_{34}$, known as the scattering elements [6]. Only three of the matrix four elements are independent, as $s_{11}^{2}=s_{12}^{2}+s_{33}^{2}+s_{34}^{2}.$ Since biological tissues are polydisperse materials, meaning they are composed of particles that are heterogeneous in size, we use a polydisperse material like IL to mimic them. Hence, these scattering elements are an important part of our simulation as they enable us to describe the polydisperse media we use in our samples [26]. To use this method, we first calculate the scattering elements for every θ by Mie scattering approximations. Calculating the scattering elements in accordance with Mie scattering you get [6]:

(8) $$\begin{array}{c} s_{11}\left(\theta \right)=0.5\cdot \left({\left|S_{2}\left(\theta \right)\right|}^{2}+{\left|S_{1}\left(\theta \right)\right|}^{2}\right)\\ \;s_{12}\left(\theta \right)=0.5\cdot \left({\left|S_{2}\left(\theta \right)\right|}^{2}-{\left|S_{1}\left(\theta \right)\right|}^{2}\right)\\ \;s_{33}\left(\theta \right)=0.5\cdot \left(S_{2}^{*}\left(\theta \right)S_{1}\left(\theta \right)+S_{2}\left(\theta \right)S_{1}^{*}\left(\theta \right)\right)\\ \;s_{34}\left(\theta \right)=0.5j\cdot \left(S_{2}^{*}\left(\theta \right)S_{1}\left(\theta \right)-S_{2}\left(\theta \right)S_{1}^{*}\left(\theta \right)\right) \end{array}$$

where the parameters $S_{1}\;\&\;S_{2}$ are the complex amplitude functions of a scattered electric field and are mainly affected by the refractive index (*n*) and the size parameter $\left(x=2\mathrm{\pi r}/\lambda \right)$, where *r* is the particle radius [20]. Since polarized light has a bivariate dependence on the scattering angles, the phase function $P\left(\theta ,\psi \right)$ with an incident Stokes vector is as follows [23]:

(9) $$P\left(\theta ,\psi \right)=s_{11}\left(\theta \right)I+s_{12}\left(\theta \right)\left(Q\cos\left(2\psi \right)+U\sin\left(2\psi \right)\right)$$

Moreover, in our experiments and in real life the scattering medium, IL and tissues, are a polydisperse material, implying that the *x* changes with each scattering event as the photon encounter a new randomized size particle according to a predetermined particle size distribution [26]. In our work, we used the distribution described by Jacques [21]. Therefore, the scattering elements change as a function of θ and x. It is important to note that *s* seen in Equation (6) is determined by the average $\mu_{s}$ of the polydisperse material and does not change according to the particle size in every scattering event [26]. Finally, after securing θ and ψ, we can update the direction vector $\hat{k}$ and the stokes parameters. Since Stokes vectors can be superimposed, all photons traveling in the direction of a detector can be added once they have been rotated properly to its plane, ensuring the reflected photons are detected properly [23].

## 3 Results and Discussion

In this section, we will discuss the results of our experiment and compare it with the simulation. The experiment and simulation consist of phantoms with different thicknesses and optical properties described in Section 2.2. While the simulation is defined by a semi‐infinite medium, the experiment is not and rather is defined by the phantom size in the petri dish. We divided our result into three main parts.

### 3.1 Normalized Intensity Reading

**FIGURE 3:**
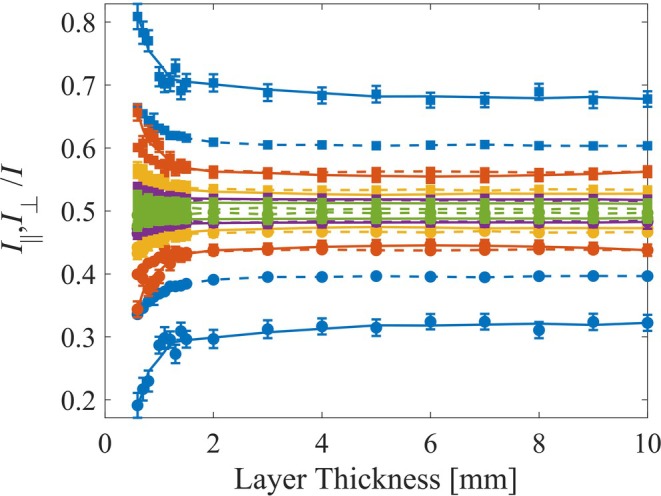
Normalized intensities for the different concentrations. The dashed line corresponds to the simulation's results while the full line correlates with the experiment. Squares mark the copolarized intensity while the circles show the cross‐polarized intensity. The colors represent the different $\mu_{s}^{\prime }$ values where blue, orange, yellow, purple and green correspond with 0.5, 1, 1.5, 2, and 3 [1/mm], respectively.

First, we compared the relationship between layer thickness and the normalized co‐polarized and cross‐polarized intensities (Figure 3). The dashed lines represent the simulation results, while the solid lines correspond to experimental data. The square marker corresponds with $I_{∥}/I$ while the circles represent $I_{⊥}/I$. The different colors indicate various $\mu_{s}^{\prime }$, with blue, orange, yellow, purple, and green representing 0.5, 1, 1.5, 2, and 3 [1/mm], respectively. For each $\mu_{s}^{\prime }$, both of the normalized intensities exhibit a converging trend toward a certain value, indicating a balance in the contributions of perpendicular and parallel components due to scattering and depolarization effects. Lower $\mu_{s}^{\prime }$ results in a larger difference between these normalized intensities, and contrariwise, seen in both experiment and simulation. For smaller thicknesses (0.6–2 mm), there is a noticeable difference in the intensity's ratios, suggesting that light is depolarized in compliance with the slab thickness.

### 3.2 Analyzing Q′‐Sensing

**FIGURE 4:**
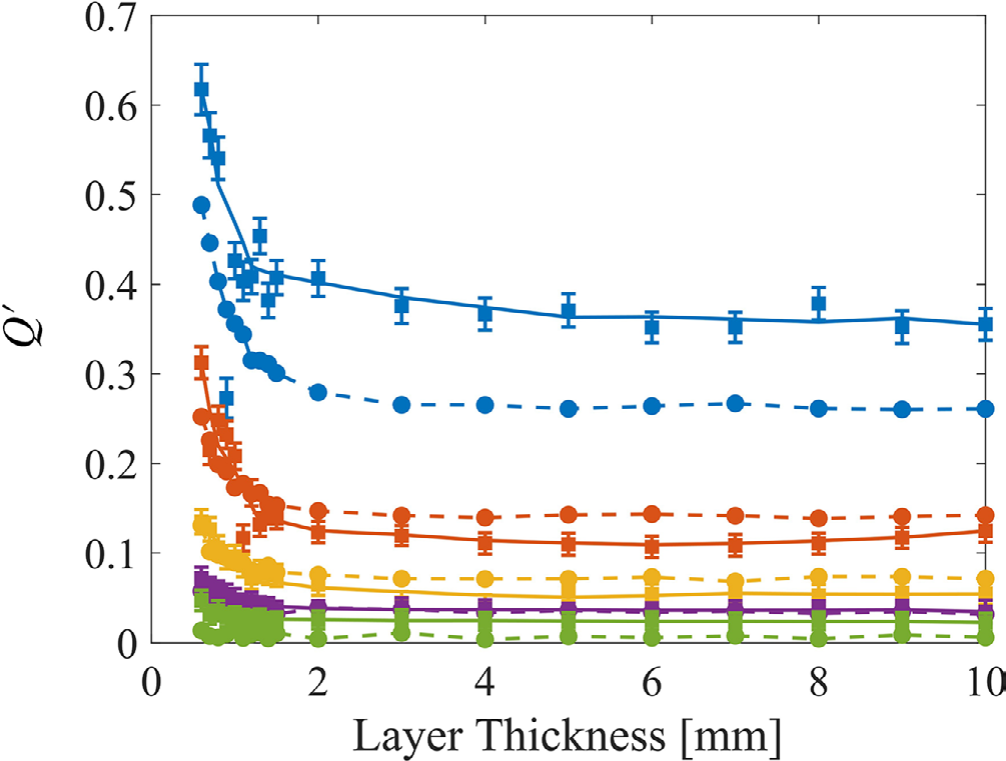
Q‐sensing for the different concentrations. The dashed line with circle markings displays the simulation's results while the full line with square markers corresponds to the experiment. The colors characterize the different $\mu_{s}^{\prime }$ values when blue, orange, yellow, purple, and green correspond with 0.5, 1, 1.5, 2, and 3 [1/mm], respectively.

Next, we present the relationship between layer thickness and the *Q*′ parameter, seen in Equation (4), for distinct scattering samples (Figure 4). As in Figure 3, different colors correspond to varying $\mu_{s}^{\prime }$ values. The graph reveals that as the layer thickness increases the *Q*′ parameter decreases, and converges to a distinct value for each scattering sample. In thin layers less than 2 mm, light retains some of its polarization before stabilizing, enhancing sensitivity when measuring reflections from superficial layers in those thickness. However, in thicker samples, the light loses its polarization, causing the *Q*′ parameter to converge, thereby enabling differentiation of optical properties based on this dependency. Furthermore, the simulation results (dashed lines) exhibit the same trend as the experimental results (solid lines).

Additionally, when performed a monodisperse simulation, where the particle size is fixed to a singular size of 200 nm (data not shown), the results differed from the IL experiments we performed (which are polydisperse). Therefore, this supports our assumption that the scatterer's size change between 20 and 700 nm was significant in the resultant scattering, as seen in the literature [21].

### 3.3 Sensing the Optical Properties

**FIGURE 5:**
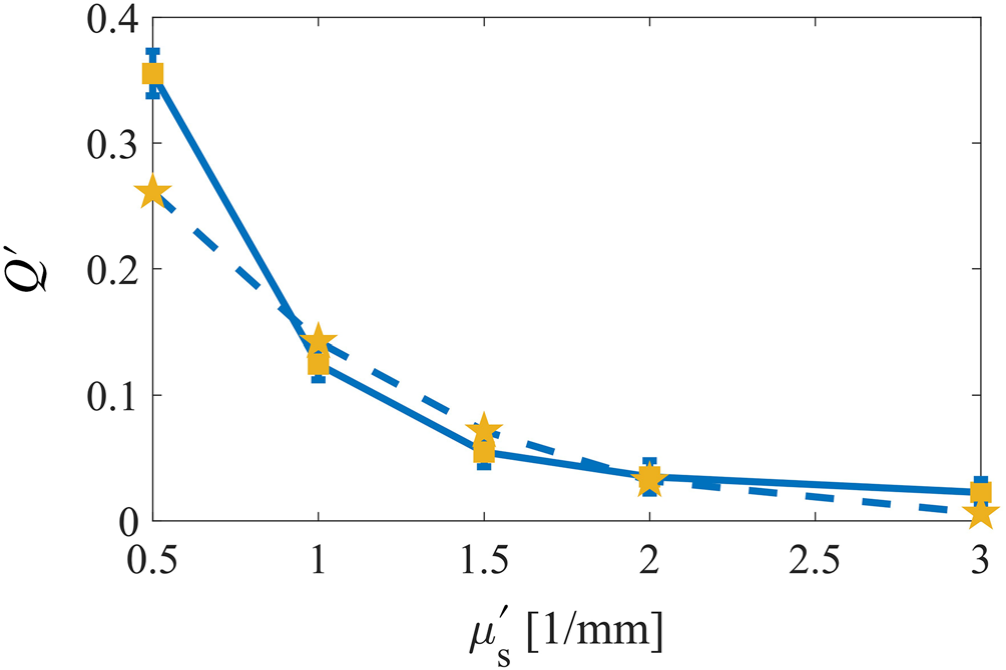
Q‐sensing of 10 mm layer thickness for varying $\mu_{s}^{\prime }$ values. The full line with yellow squares represent the experiment's results while the dashed line with star markers represents the simulation's results.

Finally, we depict the relationship between the $\mu_{s}^{\prime }$ and *Q*′ parameter for 10 mm thick samples (Figure 5). Since, the samples only differ in $\mu_{s}^{\prime }$, changes in the *Q*′ parameter solely depend on the optical properties. Hence, by measuring the *Q* parameter in thick samples where the *Q*′ parameter has converged, $\mu_{s}^{\prime }$ can be extracted.

As $\mu_{s}^{\prime }$ increases *Q*′ decreases sharply because the photons experience more scattering events before reflecting to the detector thus losing their initial polarization. The initial sharp decline suggests that small increases in $\mu_{s}^{\prime }$ have a substantial impact on depolarizing the light, making this regime optimal for the extraction of the optical properties using Q‐sensing. As $\mu_{s}^{\prime }$ approaches 3 [1/mm], the curve flattens, indicating that further increases in scattering have a diminishing effect on the polarization making it challenging to extract the correct optical properties. This trend highlights the strong dependence of light depolarization on the scattering properties of the medium and underscores the importance of $\mu_{s}^{\prime }$ in characterizing optical behavior in tissue‐mimicking phantoms. Furthermore, this method can be of great assistance to monitor the optical properties of scattering materials in laboratory conditions.

## 4 Conclusion

In this study, we demonstrated light depolarization in different tissue‐mimicking phantoms. Regardless of phantom thickness, the light stabilized at a fixed value, unique to each phantom with different scattering coefficients, as shown in Figure 4. Each curve exhibits an exponential decay to a certain value rather than zero. The light does not fully depolarize (decay to zero) when we read from the center of the illumination spot (regardless of the phantom thickness) since the $I_{s}$ remains unphased. However, we assume that the light will depolarize completely when reading at a certain offset distance from the center of the illumination. Nevertheless, this unique feature, where the light converges to a certain $I_{s}$, allowed us to extract the $\mu_{s}^{\prime }$ from the samples as $\mu_{a}$ is constant, seen in Figure 5. While this method is simple and efficient, challenges remain. First, extracting $\mu_{s}$ depends on anisotropy, necessitating the isolation of g for measurements across various materials. This is due to our lack of knowledge whether the behavior is partially attributed to anisotropy or solely the scattering. Furthermore, we assumed a negligible $\mu_{a}$, so for future work we would also need to address this issue by improving our method allowing us to isolate this parameter as well. Finally, we need to experiment with real life samples of monodisperse media to establish if there is a change compared with our results of the polydisperse IL. Nevertheless, the results demonstrate the effectiveness of using Q‐sensing to analyze the optical properties of tissue‐mimicking phantoms. It enabled the extraction of $\mu_{s}^{\prime }$ by using polarized light. In addition, the consistency between the experimental and simulation data validates the accuracy of our simulations, providing a reliable method for characterizing optical behavior in scattering media. Error that may be seen can be attributed to the changes in the IL particle size distribution in low concentrations, the efficiency of the polarizers and so forth. This approach offers significant potential for advancing the understanding and monitoring of optical properties in various materials under laboratory conditions. Knowing the penetration depth and differentiating whether we receive photons from 1 mm deep or 10 mm deep can help in various applications. For example, this feature can help in sensing the penetration of a dose of epidermal medicine to deep layers of the skin or in neurophotonics as we are interested in analyzing the photons passing through skull while reflecting from the brain. Thus, this approach can be relevant and helpful in many areas, suggesting further research should be undertaken. In our future experiments, we will be automating our translation stage allowing us to map the intensity across the *XY* plane and we will be changing the absorptivity of the medium experimented on as well to see how it might influence the tissue.

## Author Contributions

The research conceptualization was formed by D.F. In addition, D.F. with H.D. were responsible for the research supervision methodology. The sample preparation as well as the experiments was done by A.T. The investigation, optical characterization of the samples, and data analysis were done by A.T. The final validation was done by A.T. and H.D. The writing of the first draft was done by A.T. while D.F. together with H.D were responsible for the review and editing for improving the paper.

## Conflicts of Interest

The authors declare no conflicts of interest.

## Data Availability

The data that support the findings of this study are available from the corresponding author upon reasonable request.

## References

[1] V. V. Tuchin , “Polarized Light Interaction With Tissues,” Journal of Biomedical Optics 21, no. 7 (2016): 71114. 27121763 10.1117/1.JBO.21.7.071114

[2] D. Fixler , R. Ankri , H. Duadi , R. Lubart , and Z. Zalevsky , “Depolarization of Light in Biological Tissues,” Optics and Lasers in Engineering 50, no. 6 (2012): 850–854.

[3] D. Fixler and Z. Zalevsky , “In Vivo Tumor Detection Using Polarization and Wavelength Reflection Characteristics of Gold Nanorods,” Nano Letters 13, no. 12 (2013): 6292–6296. 24261467 10.1021/nl403927c

[4] I. Yariv , H. Duadi , and D. Fixler . “An Optical Method to Detect Tissue Scattering: Theory, Experiments and Biomedical Applications.” In Nanoscale Imaging, Sensing, and Actuation for Biomedical Applications XVI, eds D. V. Nicolau , D. Fixler , and E. M. Goldys , vol. 10891 (San Francisco, CA: SPIE, 2019), 1089105.

[5] H. Duadi , M. Nitzan , and D. Fixler , “Simulation of Oxygen Saturation Measurement in a Single Blood Vein,” Optics Letters 41, no. 18 (2016): 4312–4315. 27628385 10.1364/OL.41.004312

[6] J. C. Ramella‐Roman and T. Novikova , Polarized Light in Biomedical Imaging and Sensing: Clinical and Preclinical Applications (Cham, Switzerland: Springer Nature, 2022).

[7] S. L. Jacques , J. R. Roman , and K. Lee , “Imaging Superficial Tissues With Polarized Light,” Lasers in Surgery and Medicine 26, no. 2 (2000): 119–129. 10685085 10.1002/(sici)1096-9101(2000)26:2<119::aid-lsm3>3.0.co;2-y

[8] J. Qi , T. Tatla , E. Nissanka‐Jayasuriya , A. Y. Yuan , D. Stoyanov , and D. S. Elson , “Surgical Polarimetric Endoscopy for the Detection of Laryngeal Cancer,” Nature Biomedical Engineering 7, no. 8 (2023): 971–985. 10.1038/s41551-023-01018-0 PMC10427430 37012312

[9] C. Stark , C. A. C. Arrieta , R. Behroozian , B. Redmer , F. Fiedler , and S. Müller , “Broadband Polarimetric Glucose Determination in Protein Containing Media Using Characteristic Optical Rotatory Dispersion,” Biomedical Optics Express 10, no. 12 (2019): 6340–6350. 31853403 10.1364/BOE.10.006340 PMC6913393

[10] T. Liu , S. Yu , X. Zhu , et al., “In‐Situ Detection Method for Microplastics in Water by Polarized Light Scattering,” Frontiers in Marine Science 8 (2021): 739683.

[11] R. Ankri and D. Fixler , “Gold Nanorods Based Diffusion Reflection Measurements: Current Status and Perspectives for Clinical Applications,” Nanophotonics 6, no. 5 (2017): 1031–1042.

[12] S. L. Jacques , Origins of Tissue Optical Properties in the UVA, Visible, and NIR Regions, Advances in Optical Imaging and Photon Migration (Portland, OR: Optica Publishing Group, 1996), OPC364.

[13] V. Kodach , D. Faber , J. Van Marle , T. Van Leeuwen , and J. Kalkman , “Determination of the Scattering Anisotropy With Optical Coherence Tomography,” Optics Express 19, no. 7 (2011): 6131–6140. 21451637 10.1364/OE.19.006131

[14] I. S. Martins , H. F. Silva , E. N. Lazareva , et al., “Measurement of Tissue Optical Properties in a Wide Spectral Range: A Review,” Biomedical Optics Express 14, no. 1 (2023): 249–298. 36698664 10.1364/BOE.479320 PMC9841994

[15] I. Feder , H. Duadi , M. Fridman , T. Dreifuss , and D. Fixler , “Experimentally Testing the Role of Blood Vessels in the Full Scattering Profile: Solid Phantom Measurements,” Journal of Biomedical Photonics & Engineering 2, no. 4 (2016): 40301.

[16] S. L. Jacques , J. C. Ramella‐Roman , and K. Lee , “Imaging Skin Pathology With Polarized Light,” Journal of Biomedical Optics 7, no. 3 (2002): 329–340. 12175282 10.1117/1.1484498

[17] S. L. Jacques , S. Roussel , and R. Samatham , “Polarized Light Imaging Specifies the Anisotropy of Light Scattering in the Superficial Layer of a Tissue,” Journal of Biomedical Optics 21, no. 7 (2016): 71115. 27165546 10.1117/1.JBO.21.7.071115 PMC4861869

[18] S. L. Jacques , “Polarized Light Imaging Sensitive to 100 to 300nm Size Range of Light‐Scattering Tissue Structure,” in Optical Biopsy XXII: Toward Real‐Time Spectroscopic Imaging and Diagnosis (San Francisco, CA: SPIE, 2024), 4–7.

[19] D. W. Hahn , Light Scattering Theory (Gainesville, FL: Department of Mechanical and Aerospace Engineering, University of Florida, 2009).

[20] C. F. Bohren and D. R. Huffman , Absorption and Scattering of Light by Small Particles (New York: John Wiley & Sons, 2008).

[21] H. J. Van Staveren , C. J. Moes , J. van Marie , S. A. Prahl , and M. J. Van Gemert , “Light Scattering in Lntralipid‐10% in the Wavelength Range of 400–1100 nm,” Applied Optics 30, no. 31 (1991): 4507–4514. 20717241 10.1364/AO.30.004507

[22] L. Wang , S. L. Jacques , and L. Zheng , “MCML—Monte Carlo Modeling of Light Transport in Multi‐Layered Tissues,” Computer Methods and Programs in Biomedicine 47, no. 2 (1995): 131–146. 7587160 10.1016/0169-2607(95)01640-f

[23] J. C. Ramella‐Roman , S. A. Prahl , and S. L. Jacques , “Three Monte Carlo Programs of Polarized Light Transport Into Scattering Media: Part I,” Optics Express 13, no. 12 (2005): 4420–4438. 19495358 10.1364/opex.13.004420

[24] I. Lopushenko , O. Sieryi , A. Bykov , and I. Meglinski , “Exploring the Evolution of Circular Polarized Light Backscattered From Turbid Tissue‐Like Disperse Medium Utilizing Generalized Monte Carlo Modeling Approach With a Combined Use of Jones and Stokes‐Mueller Formalisms,” Journal of Biomedical Optics 29, no. 5 (2024): 52913. 10.1117/1.JBO.29.5.052913 PMC10715447 38089555

[25] S. A. Prahl , “A Monte Carlo Model of Light Propagation in Tissue, Dosimetry of Laser Radiation in Medicine and Biology,” Dosimetry of Laser Radiation in Medicine and Biology 10305 (1989): 105–114.

[26] J. C. Ramella‐Roman , S. A. Prahl , and S. L. Jacques , “Three Monte Carlo Programs of Polarized Light Transport Into Scattering Media: Part II,” Optics Express 13, no. 25 (2005): 10392–10405. 19503254 10.1364/opex.13.010392

